# Barriers to Creating and Implementing Child Mental Health Policies That Promote Child Mental Health Competence in Victoria, Australia: A Qualitative Study

**DOI:** 10.1002/hpja.70046

**Published:** 2025-04-30

**Authors:** M. Stonnill, S. Gray, S. Woolfenden, S. Goldfeld

**Affiliations:** ^1^ Centre for Community Child Health Murdoch Children's Research Institute Melbourne Australia; ^2^ Department of Pediatrics University of Melbourne Melbourne Australia; ^3^ Community Paediatric Research Group Sydney Medical School, University of Sydney Sydney Australia; ^4^ Sydney Institute for Women, Children and Their Families, Sydney Local Health District Sydney Australia

**Keywords:** child wellbeing, family support, health policy

## Abstract

**Introduction:**

A comprehensive child mental health policy agenda should promote mental health competence and prevent difficulties to ensure children experience the best start to life. As a key influence on child outcomes, supporting families is critical to achieving this. This study aims to understand the current child mental health policy landscape from a policy maker's perspective. It explores barriers to creating and implementing policies that support families and target child mental health competence for children aged 0 to 12 in Victoria and nationally.

**Methods:**

Eleven policy makers from health, education, social services/human rights, and non‐government organisations participated in semi‐structured interviews. Interviews were de‐identified and analysed using the general inductive approach outlined by Miles et al.

**Results:**

Policy makers described the current child mental health policy landscape as focused on addressing mental health difficulties. This prioritisation of addressing difficulties and family risk factors, along with challenges associated with measuring competence and the complex mental health system, was seen as hindering the implementation of child mental health competence policies and their ability to support families.

**Conclusions:**

While there are efforts to promote child mental health competence through policy, such as the National Children's Mental Health and Wellbeing Strategy, policy makers believe there remains a strong focus on deficits. **So What?** Researchers can play a role in addressing barriers to creating mental health competence policies by providing guidance on the measurement of child mental health competence and identifying ways to stack interventions to efficiently target mental health competence and difficulties and address family risk factors.

## 1 Introduction

Optimal mental health is not just the absence of mental health difficulties, but also the experience of mental health competence, sometimes called wellbeing, positive mental health, or thriving [1]. Mental health competence describes success in developmentally appropriate tasks [2] such as social competence, emotional regulation, and prosocial behaviours [1]. These competencies, as well as mental health difficulties, have an ongoing impact on learning, behaviour, and health outcomes during childhood, and into adolescence and adulthood [3, 4, 5, 6]. Mental health competence changes over time, as mental health competence is not fixed; there is opportunity to intervene early to address the factors that influence mental health competence outcomes [1].

Children's mental health is shaped by a wide range of environments in which they learn, live and play—known as the social determinants of health [7, 8]. For example, early childhood education has a positive effect on competencies such as readiness to learn [9], neighbourhood green spaces are associated with reduced emotional and behavioural difficulties in children [10], and the instability in care and lack of access to mental health support for children in out‐of‐home care can result in poorer mental health outcomes [11]. Risk and protective factors in the family environment are particularly key in influencing child mental health competence and difficulties [12, 13, 14, 15]. Family risk factors including parent mental health difficulties and parenting behaviours are associated with child mental health difficulties [16, 17, 18, 19], and family protective factors including parenting self‐efficacy, parent–child relationships and emotional regulation are associated with the development of competencies [12, 13, 14].

To help children thrive we therefore need policies and interventions that both prevent and reduce mental health difficulties [20], but also promote mental health competencies, this is consistent with the WHO definition of health as “a state of complete physical, mental and social wellbeing and not merely the absence of disease or infirmity” [21]. Currently there are a broad range of policies within Australia that both aim to reduce mental health difficulties for children, for example the Head to Health Kids National Service Model [22], and promote competence and thriving, such as the National Children's Mental Health and Wellbeing Strategy [23]. Broadly, these policies target child mental health in two ways: [1] through mental health service provision‐ targeting mental health difficulty need‐ and [2] through universal school and early childhood policies and interventions‐ targeting mental health promotion and early identification of difficulties. The Royal Commission into Victoria's Mental Health System identified a gap in mental health services for children 0–12 years [24]. In response there has been an increased push to provide services for this age group. In Victoria this is reflected in the establishment of three Children's Health and Wellbeing Locals which aim to deliver specialist medical and allied health services for children aged 0–11 and their families [25]. There is also work to address this gap at a national level through the Kids Head to Health Hubs [22]. As universal systems, early childhood and primary schools have been leveraged to target prevention and early intervention initiatives, the Victorian Mental Health in Primary Schools program provides funding to employ a mental health and wellbeing leader in all government and low‐fee non‐government schools [26], and the Schools Mental Health Fund and Menu provides Victorian schools with a selection of programs to support students mental health and wellbeing [27]. At a national level, the Australian Student Wellbeing Framework provides schools with a set of guiding principles to support them build positive learning environments [28]. While there is a broad range of policies targeting child mental health in Australia, it appears to be less common for policies to focus on families as a way to influence child mental health outcomes. As a critical influence on the mental health outcomes of children, families should be considered alongside education and service provision when creating and implementing child mental health policies and interventions.

Given the wide range of risk and protective factors across sectors, it is unlikely that a single intervention or policy will improve the mental health competence of children. Additionally, there is evidence that exposure to more than one protective factor, such as more than one early years service (e.g., parenting programs, antenatal care, early childhood education), has a positive, cumulative effect on child development [29], and exposure to multiple protective factors can act as a buffer to the negative effects of exposure to multiple risk factors [30]. Therefore, to improve outcomes for children, we need a cross‐sectoral approach that considers how to stack and promote policies and interventions that target child mental health competence across multiple disciplines.

To understand the current child mental health policy landscape from a policy maker perspective, this study aimed to explore barriers to creating and implementing policies that target child mental health competence for children aged 0 to 12 in Victoria and nationally. Additionally, we explored policy makers perspectives on the barriers to supporting families as a mechanism to promote competence.

## 2 Methods

### 2.1 Selection and Recruitment Process

Purposive snowball sampling was used to identify potential participants. Participants were purposively sampled from an existing knowledge and translation group made up of policy experts from Australian state and federal governments, and non‐government organisations, as well as author existing networks. Potential participants were invited to participate if they had involvement in the creation, implementation, or oversight of child policies in Victoria or nationally. While there is work occurring across all Australian States and Territories, the interviews targeted Victorian policymakers as an exemplar in the context of the recent Royal Commission into Victoria's Mental Health System and increased spending on child mental health initiatives in schools [24, 27]. Twenty‐eight potential interviewees were invited to participate; 11 participants agreed to take part in the study in accordance with the protocols approved by The Royal Children’s Hospital Melbourne Human Research Ethics Committee [HREC/96769/RCHM‐2023].

### 2.2 Interview Schedule and Process

A semi‐structured interview protocol was developed by the research team to answer the study aims, including (1) describing the current policy environment, (2) identifying barriers to creating and implementing policies related to competence, (3) identifying barriers policymakers face to supporting families as a mechanism to promote child mental health competence, and (4) describing the future of child mental health policy (Appendix A). This approach allowed us to focus the interviews around pre‐established questions, while also allowing interviewees to bring up and comment on other relevant themes as the interview progressed [31]. Using this approach ensured the key aims of the study were achieved by guiding the discussion, while also gaining greater breadth of responses from interviewees, which was considered important as interviewees worked across different jurisdictions.

Interviews took place between October 2023 and July 2024. Participants were provided with a plain language statement and implied consent by scheduling an interview. Participation involved a semi‐structured interview lasting no longer than 60 min, scheduled at a time convenient to the participant and conducted via video‐conferencing.

All interviews were audio recorded and transcribed using Microsoft transcribe; transcripts were checked by the interviewer (MS) for accuracy before analysis.

### 2.3 Data Analysis

Interview analysis was guided by the general inductive approach outlined by Miles, Huberman and Saldana [32]. Interview analysis had three concurrent activities:

1. Data condensation: Raw data was condensed through a process of selecting, focusing, simplifying. Initial condensation was guided by the key research questions and the semi‐structured interview questions. Condensation occurred continuously through the analysis; early coding of raw text was descriptive to provide an overall sense of what the data contained. This was followed by inferential coding to identify patterns in the responses from interviewees. Coding was iterative, checking for missing information and ensuring themes are coherent and reflective of the data.
2. Data display: Where appropriate data displays have be used to summarise key themes from the analysed data, displaying relationships between ideas. Data displays aim to make the information more accessible and are considered a part of the analysis process.
3. Drawing and verifying conclusions: Conclusions were drawn from the analysed data; this process occurred concurrently with coding and creating displays.

NVivo 14 was used to code data.

## 3 Results

Policy makers were primarily from Victoria and represented health, education, and local government departments (Table 1). There were two policy makers from commonwealth social services/human rights departments and two policy makers from non‐government organisations who held national perspectives.

**TABLE 1:** Interview participants.

Department	Abbreviation	Number of participants
Victorian health departments	VH	4
Victorian education departments (state and regional)	VE	2
Victorian local government peak body	VL	1
Commonwealth social services/human rights	AS	2
Non‐government organisations	O	2

Key themes identified during analysis are presented in Table 2. Thirteen key themes were identified and are grouped under categories relating to the aims of the study.

**TABLE 2:** Key themes.

Category	Key theme	Theme description
Current policy environment	Focus on deficits	Current policy environment is characterised by a focus on addressing mental health difficulties rather than competencies
	Competencies built into the education system	There are competencies built into the education system through social and emotional curriculum goals
Barriers to targeting child mental health competence	Siloed nature of government	Large and siloed nature of governments prevents communication and collaboration
	Need to address existing difficulties	It is important to target existing mental health difficulties, the prioritisation of resources to address difficulties can erode efforts to promote competence
	Difficult to measure competence	Without clear measurements it is difficult to demonstrate impact and gain support for mental health competence policies
	Difficult to incorporate the voice of the child	Child perspective is needed to inform successful policies and services
Barriers to supporting families to improve child mental health outcomes	Complicated mental health system	Navigating the mental health system, including different levels of the system and referral pathways, can be a burden to families
	Complex needs and stress of families	The challenges families face need to be addressed before they can be supported to promote competence
	Family mental health literacy	Families need a degree of mental health literacy to know when to seek help and to promote competence
	Lack of training and capacity building for professionals working with families	There is a skill gap in mental health policy makers and practitioners in how to work with families, and in some cases understanding competence
Future of child mental health policy	Continued support for and increase in early intervention	Continuation of current work focusing on the prevention and early intervention for mental health difficulties
	Increased holistic view of wellbeing and strengths‐based approaches	Improved understanding of wellbeing and incorporation of strength‐based approaches
	Waning interest in child mental health	Government financial pressures resulting in the prioritisation of other areas

### 3.1 The Current Policy Environment

Policy makers across all jurisdictions identified a focus on deficits and addressing difficulties in the current policy environment; however, this focus on deficits was particularly prevalent in the health space:

I think the policy environment in health is very focused on prevention of illness and treatment, more at the sort of treatment end and less focused on prevention, and in terms of promotion of competence, I think it's almost non‐existent. (O2)

This deficit mindset was described as permeating the whole system, from clinicians to policy makers:

We have a collective mindset of a deficit mindset and a deficit focused approach. That is more than just the clinicians and the practitioners who work directly with children. That is, tends to be, the policymakers and decision makers, and anyone who is working indirectly in children's mental health space as well. So that attitude and belief is impacting on the policies and the practices and what the children and the families are experiencing. (VL1)

Some policy makers saw this focus on deficits as a disconnect between current policies and practice:

I suppose I had strong hopes for the Royal Commission [into Victoria's Mental Health System] and the recommendations and the sad reality from my lens hasn't quite met that. (VH4)

While policy makers from education departments believed that there was a focus on deficits, they also identified that in education, competencies are built into the system through the provision of social and emotional curriculum goals:

It's well built into our system, that building those capabilities is an outcome of strategic importance and is schools core business. And in terms of how well that's done, I think it's done very well. But to your earlier point, sometimes a lot of the focus of that effort is more around problems than around strengths and broader capabilities and I think that there's variable practice and that we're still early in some of those objectives. (VE1)

### 3.2 Barriers to Targeting Child Mental Health Competence

In relation to barriers to targeting competence, the most common theme to emerge from the interviews was the siloed nature of governments. Policy makers from across jurisdictions believed large and siloed governments prevented communication and collaboration within and between government departments, which were seen as necessary for this work:

We're not great at doing joined up government work and children and families requires that, prevention or promotion requires that as well… It's really hard to do joined up work and that's not just across government, but also across the sector (VH3)

The need to address difficulties and the prioritisation of funding to addressing those difficulties also emerged as a key barrier to targeting competence. Previous efforts to promote competence and resilience were seen as eroded by the competing needs of addressing difficulties:

I think the model got diffused and partly that was because of the pressure of the clinical demand and it took the focus off. So it was seen, I guess, a bit like a seesaw, mental health promotion, prevention, early intervention was a bit like a luxury when you're drowning with the direct referrals. (VH4)

Policy makers also discussed difficulties in the creation and monitoring of policies related to child mental health competence, including measurement and incorporating the voice of the child. Compared to mental health difficulties, policy makers felt it was difficult to measure and demonstrate impact for policies that target mental health competence. The difficultly in measuring impact was seen as making mental health competence policies politically unviable:

my concern is…, that whole competency and intervention and identification before that even, the health promotion side of things, the teaching of parents about how to actually start spending time and focusing on what can be positive about that to prevent those things, I think the problem we have with demonstrating impact of any of those things is that it it's difficult to measure the absence of something, and so it doesn't get the attention in the political realm because what's the measure? (VL1)

Difficulties associated with accessing the perspectives of children to inform policy and services was seen as preventing high quality policies targeting children, however there was conflict between policy makers regarding what this looked like. One view was that there were bureaucratic and legal constraints to being able to hear the voice of the child:

the voice of the child, there are a lot of barriers to hearing that, as there should be, so for example there are legislative and safety requirements about how we engage with people under the age of 18. And certainly the 0‐11's is very difficult to really put them at the centre, to hearing their own voice, and hearing their own needs (VH1)

However, there was not complete agreement with some noting that the problem sat more with policy makers and a lack of genuine interest in the voice of the child. Despite opportunity to listen to the voice of the child, where consultations with children have occurred, they haven't been translated into policies, with the concerns of adults dominating policies:

there are now far more opportunities to listen to children. Policy makers just need to make that a priority I think to do that. But having said that, there are many more opportunities. What I haven't seen is what the kids say being turned into policy. So we're not doing that translation into policy… we need to stop asking kids the same questions again and again. We need to start acting on what they've told us before and going back to them and saying here's what we did with what we heard from you (AS2)

### 3.3 Barriers to Supporting Families to Improve Child Mental Health Outcomes

Policy makers identified both barriers in the mental health system that hindered providing support for families and existing barriers at a family level that impacted their ability to promote mental health competence.

At a system level, a lack of training and capacity building was identified as a key barrier to supporting families and promoting child mental health competence. In the education system, this gap was described as a gap in understanding of child mental health competence and mental health difficulties. However, in the health and social services/human rights areas, there was a gap in practitioners trained to work with families, with some practitioners seen as stuck in an individualised model of care without considering the child in the context of the family:

[practitioners who] predominantly come from an adult model who had never worked with families… in that very basic level have to be skilled up to have the way to actually work with the family and sit in the room with the family (VH4)

The complicated mental health system plagued by a lack of coordination was seen as a key barrier for families to access support for children and themselves:

families can be dealing with an array of complexities in their own home like which can make accessing services difficult, because you have perhaps a web of services that you are linked into and there might be a lack of coordination of those services. I think there is quite possible difficulty navigating a complex system, and what I mean by that is knowing who to ask about what and there not being a high level of coordination of these things (VH1)

Linked closely with the complexity of the mental health system was the complex needs and stress of families. Policy makers saw the complex needs of families, and the need to address these challenges first, as preventing some of the work to promote mental health competencies:

families are becoming more and more complex, there's more and more going on for them and so actually getting to do some of that really positive work… the opportunity for that, I guess is a bit less, particularly for those families that are more vulnerable (VL1)

The importance of families having good mental health literacy for both the identification and treatment of mental illness, as well as the promotion of ‘good’ mental health, was identified as a key factor in families being able to take a proactive role in their child's mental health competence:

being that proactive, to do that they need that understanding of what is normal mental health and what does good mental health look like and what is mental health that needs support look like (VL1)

### 3.4 The Future of Child Mental Health Policy

Looking forward, most policy makers expressed positive views of the future of child mental health policy. There was a belief by some that there would be continued support for and an increase in early intervention for mental health difficulties. In Victoria specifically, policy makers from both health and education believed there would be continued focus on implementing the recommendations of the Royal Commission into Victoria's Mental Health System. There was also a belief that there would be an increased holistic view of child wellbeing, including an increased understanding of the social determinants of health, an increase in strengths‐based approaches in the education system, and a shift from therapeutic models of care to a greater focus on wellbeing:

I think that we are moving in the right direction in the way that people are seeing the need to have that macro view to have that more holistic understanding about wellbeing. But we still need to be zooming in, I'm not saying we can't be looking at only mental health, but we need to be looking at mental health within the context of holistic wellbeing. And so I think we are moving in that direction ever so slowly (O1)

While the overall view was positive, there was some disagreement about the future of child mental health policy. A couple of policy makers who held key leadership positions felt there was waning interest in child mental health:

I think we went through a bit of a wave where mental health was really getting on everybody's agenda and particularly the politicians' agenda and the communities' agenda. I think now we're under such dire pressures financially in our society, and homelessness and all the other pressures, mental health is being taken off the screen really. And so I'm a bit pessimistic about, particularly early interventions, so for me, early intervention is everything to do with children, that's where we should be intervening, and all the research shows that's the most effective use of the dollars if you're really wanting to change people's mental health (VH4)

Without a renewed and targeted focus on child mental health policy, this slipping in the prioritisation of child mental health was seen as unlikely to change:

I think there was quite a lot of attention after the pandemic and then we had that inquiry into school refusal and, to some extent, there's still discussion about those things, the effect of the pandemic, but I think it's waning. I think the attention is sliding away, and short of another crisis kind of coming on, policy tends to be driven by crises, this is the truth of it, I feel that without some focused attention and an advocacy level amongst a broad group of advocates and stakeholders, I don't know that by itself it will improve (AS2)

## 4 Discussion

This study described the current child mental health policy landscape from a policy maker perspective and identified the key issues in relation to the barriers in implementing policies focused on mental health competence and supporting families. Policy makers identified a deficit mindset, focused on addressing mental health difficulties, in the current child mental health policy landscape across health, education, and social service departments in Victoria and nationally. This deficit mindset, along with key structural issues, such as a lack of coordination between services, was seen as hindering the implementation of child mental health competence policies and their ability to support families. Additionally, the continued need to prioritise and address mental health difficulties was seen as hindering progress towards addressing competence.

### 4.1 Current and Future Policy Landscape

To achieve optimal mental health for children, we need to address mental health difficulties and promote competencies at the same time [1]; this is reflected at both a national and a state level through policies that promote child mental health competencies separate to or alongside the prevention and treatment of child mental health difficulties [23, 28, 33, 34]. Despite the existence of policies targeting competence, policy makers characterised the current policy landscape as focused on deficits and addressing difficulties. This may indicate that these strengths‐based policies are not being implemented to their full potential. While there is an increasing recognition that wellbeing is separate from the absence of disease and there has been a shift from a focus on mental health difficulties and diagnosis towards supporting positive mental health over the last few decades [35], the views of policy makers indicate that there is still a way to go to shift this deficit mindset.

Most policy makers held a positive perception of the future of child mental health competence policies. However, without addressing the current disconnect between policy aspirations and the perceptions of policy makers, it will be difficult to realise this view of the future.

### 4.2 Competing Needs of Mental Health Difficulties

While there is a need to target mental health competence through policy, there is also continued need to address mental health difficulties. In Australia, 14% of 4–17‐year‐olds experience a mental health disorder [36]; however, the majority of Australian children with mental health disorders aren't getting professional help [37]. This gap in appropriate services for children 0–12 years old was also highlighted during the Royal Commission into Victoria's Mental Health System [25]. In this study, the interviews identified the need to address the existing mental health difficulties of children as a competing priority for policy makers attempting to target mental health competence. When there is still a great need to address mental health difficulties, it is hard to prioritise resources to support the promotion of mental health competence, and in some cases even the prevention of mental health difficulties.

Children experience the best outcomes if they are both free from mental health difficulties and experience high mental health competence [38]; therefore, if we want to support children to thrive, it is not sufficient to only target difficulties. In addition to the challenge of targeting both competence and difficulties, policymakers in this study highlighted that many families have complex needs that need to be addressed before they can be supported to promote their child's mental health competence. They also identified that the siloed nature of government often hinders their work. This underscores the complexities in trying to improve child mental health competence and that it cannot be achieved in isolation. Researchers can play a role in helping policymakers address these complex challenges, such as by providing evidence for precision policy and ‘stacking’.

To support policy makers more effectively allocate resources so they can address both competence and difficulties, researchers should generate evidence that is specific and considers factors such as which policies to implement, when and to whom [39]. This precision in evidence will enable more targeted and effective policy responses, ensuring resources are allocated efficiently to address both competence and difficulties [39]. In addition to generating evidence for precision policy, researchers can identify opportunities to ‘stack’ policies, to enhance their impact and optimise use of resources. Evidence shows that stacking interventions, combining multiple evidence‐based interventions, has a positive impact on child outcomes [29, 30]. Taking into account common factors influencing competence and difficulties, such as maternal mental health [16, 17, 40], stacking interventions could be one strategy to address difficulties and promote competence simultaneously. It can also be used to address the complex needs of families and presents a way to combine efforts across sectors. Precision policy and stacking were not identified in the interviews as strategies to reduce barriers such as the difficulty in addressing mental health competence alongside mental health difficulties, or the complex needs of families. However, policy makers recognise these complexities and the need for strategies to address them, this is evident through cross‐sector policies such as the Early Years Strategy [41]. Precision policy and stacking represent opportunities for researchers to contribute to the improvement of child mental health policies in the future by identifying strategies to use limited public funding in ways that are targeted and as effective as possible [42]. With the hope that this more efficient use of resources will allow policy makers to address both competence and difficulties.

### 4.3 Structural Issues Hindering Support for Families

Policy makers identified structural issues such as navigating a disjointed and complex system, as well as a lack of training relating to families, as key issues in their ability to support families. This is consistent with research into the care of children with complex mental health conditions which found that health care system factors such as availability and quality of providers and silos including lack of coordination between sectors were considered barriers to accessing optimal care [43].

The Royal Commission into Victoria's Mental Health System also highlighted some of these structural barriers in accessing support for mental health difficulties. For example, conflicting intake requirements can contribute to a disruption in treatment, care and support, as children move between services and need to retell their experience or are faced with practitioners that don't meet their needs [44].

The barrier of the complex mental health system is further compounded by the complex needs of families, which need to be addressed for children to receive the best outcomes. As a key influence on child mental health competence and difficulties, it is imperative policies address family risk factors and promote family protective factors to ensure children have the best possible mental health outcomes [12, 13, 14, 15]. It is promising that within the Australian primary health care system there is currently an appetite for policies that address family adversity [45], as without efforts to address these barriers it is unlikely that policies to leverage family strengths and promote child mental health competence will have much traction.

### 4.4 The Need for Good Quality Data

Promoting optimal child mental health outcomes (high competence and low difficulties) is also reliant on good quality data [8]. Challenges associated with monitoring and evaluating were identified by policy makers as a barrier to creating and implementing child mental health competence policies. Current data and information gaps in national reporting limit the extent we can understand child wellbeing, including mental health competence [46]. Work has begun to enhance the measurement of child‐level disadvantage to improve understanding of the extent and key drivers of child disadvantage, supporting policy makers in their decision making [47]. Additionally, enhancements to existing administrative data sources (such as data collected through schools and health services) have been proposed to fill data collection gaps [46]. To ensure mental health competence does not continue to fall behind, efforts to improve child‐centred data collection should include measures of child mental health competence. Without the ability to measure policy impact, policies targeting mental health competence will remain unviable. Going forward, developing purpose‐built scales that can be incorporated into national longitudinal and administrative data collection will be important for monitoring population prevalence of mental health competence, ensuring efforts to promote competence are effective.

This work is further complicated by overly broad definitions of wellbeing [48] and a lack of a gold standard of measurement of mental health competence. A scoping review of child wellbeing measurement in Australia identified several existing national frameworks and indicator sets relevant to the measurement of children's wellbeing [49], consistent with the broad nature of wellbeing, these measures occurred across several domains including health, social support, justice and safety, housing, and education. When narrowing our focus to mental health competence, there remains a gap in measures to help us understand this key component of overall child mental health. Measures of competence have been derived from existing measures, such as using items from the Strength and Difficulties Questionnaire [1, 50], this is limited as it is not purpose‐built, and measures such as the positive mental health scale are focused on adults, not children [49].

### 4.5 Limitations

While interviewees were primarily from Victoria, there is work occurring across all Australian states and territories related to child mental health competence, such as the New South Wales Brighter Beginnings [51], Queensland's Putting Queensland Kids First [52], and the South Australian Royal Commission into Early Childhood Education and Care [53]. Therefore, these findings may be useful for other policy makers outside the Victorian context.

While there was some overlap in the views of education, social/human rights, and health policy makers, many of the barriers identified more closely related to the health system compared to other systems. This might reflect an ongoing perception of mental health as a responsibility of health departments; however, it may also reflect the uneven distribution of interviewees from a health background (including individuals from local and non‐government organisations).

## 5 Conclusion

While there are efforts to promote child mental health competence through policy, such as the National Children's Mental Health and Wellbeing Strategy, from the perspective of policy makers there remains a strong focus on deficits. Without prioritising and stacking policies targeting both mental health competence and mental health difficulties, we will be unable to improve optimal child mental health in the future; this is important because anything less than optimal mental health is associated with worse outcomes for children [20]. Supporting families is essential to promoting the mental health competence of children; however, structural issues within the mental health system, often compounded by family risk factors, are preventing policy makers from effectively supporting them. Researchers can play a key role in helping to address the challenges faced by policy makers by advocating for a dual model of mental health, providing guidance on the measurement of child mental health competence outcomes, and identifying ways we can stack interventions to efficiently target both mental health competence and difficulties, and address family risk factors. By addressing these implementation barriers we can more effectively support families and promote mental health competence, ensuring children have the best possible mental health outcomes.

## Ethics Statement

Ethics approval was granted by The Royal Children's Hospital Melbourne Human Research Ethics Committee [HREC/96769/RCHM‐2023].

## Conflicts of Interest

The authors declare no conflicts of interest.

## Data Availability

Research data are not shared.

## Appendix A Semi‐Structured Interview Protocol

**Table:**

Question	Prompts
0. Can you start by telling me a little about where you work and your role.	
How well do you think the current policy environment supports the mental health competence of children?	a. What are the main mechanisms through which child mental health competence is targeted? e.g., school‐based, support for families, neighbourhood level interventions, primary health care
2 What are the current barriers to creating and implementing policies targeting mental health competence in children?	a. Think about both barriers to creating policies targeting children 0–12 years (compared to adolescents) and barriers to targeting mental health competence (compared to mental health difficulties).
3 What are the current barriers to supporting families as a mechanism to influence child mental health competence?	a. What are the barriers for families to access support provided through current policies and services?
	b. Are there any barriers specific to the experience of different types of families, e.g., rural, Cultural and Linguistically Diverse?
4 What are the current barriers to measuring the success of policies targeting mental health competence or families?	a. Are there any other barriers we haven't discussed that you would like to mention?
5 Where do you see the child mental health policy landscape in 5 years?	a. Will mental health difficulties or mental health competence or both be the focus?
	b. What mechanisms will be targeted to influence child mental health?
6 What will be the key drivers of future child mental health policies/what will determine the future policy landscape? e.g., research, advocacy, following the status quo, internal department structures etc.	
7 What can researchers do to ensure findings are translated into policy?	
8 My final question is about language, I have focused on competence, but what would be the most common language used in the department to describe positive child mental health? Do you think research should match this language to make it clearer what we are targeting and support translation into practice?	
9 Do you have any other comments?	a. Is there anyone else you could recommend I speak to?
